# The Journey of an Outstanding Scientific Mind: Prof Hiroshi Maeda (1938–2021)

**DOI:** 10.3390/jpm11121362

**Published:** 2021-12-14

**Authors:** Khaled Greish, Jun Fang

**Affiliations:** 1 Princess Al-Jawhara Center for Molecular Medicine, Department of Molecular Medicine and Nanomedicine Unit, College of Medicine and Medical Sciences, Arabian Gulf University, Building 61, King Abdulaziz Avenue, Manama 328, Bahrain; 2 Faculty of Pharmaceutical Sciences, Sojo University, Ikeda 4-22-1, Nishi-ku, Kumamoto 860-0082, Japan

In the mid-70s of the last century, Prof. Maeda and colleagues were studying the antibiotic protein neocarzinostatin (NCS) as part of his work in the Department of Microbiology, Kumamoto University Medical School in Japan. Prof. Maeda’s early work showed that the potent antitumor protein was inactivated in the serum or by cell homogenate through proteolysis. This was the prelude for the invention of the first approved nanomedicine in the world (SMANCS) in 1991 [1]. Soon after its invention and regulatory approval in Japan, SMANCS was recognized as the most effective treatment against hepatocellular carcinoma [2]. This invention was the forefront of a long list of currently approved nanomedicines that are used in the clinic for cancer treatment as well as other conditions.

The visionary mind of Prof. Maeda conceived personalized medicine in management of liver cancer patients in his practice. The dose of SMANCS in lipiodol was personalized and tailored for each patient based on the size of the tumor, the uptake, and retain of SMANCS. In Hakoaiki hospital, Kumamoto, Japan, Prof. Maead’s group used to ascribe patients to different doses from 1× to 4× SMANCS lipiodol based on the system devised by Prof Maeda.

SMANCS was amphiphilic drug that is freely soluble in the lipiodol. Upon injection, the drug can be visualized as radiopaque concentration in liver mass through X-ray. The patients were followed up to see the uptake of the drug as well as its retainment over 3 weeks. This system was a perfect application of what came to be known as theranostic. Prof. Maeda’s group studied extensively not only the effect of nanomaterials on the cancer cells, but also on cancer as a tissue [3]. He identified the effect of angiotensin II, nitric oxide (NO), among other key players to govern the selective concentration of SMANCS in hepatocellular carcinoma patients. Further to his innovative ideas and diligent studies, the application of SMANCS in lipiodol in adjunction with NO donors and angiotensin II was used to enhance further SMANCS concentration in HCC patients [4]. Once more, Prof. Maeda was ahead of the mainstream scientific thinking, and directly utilizing it to take excellent care of patients.

During the research of SMANCS, Prof. Maeda and his colleague discovered the famous enhanced permeability and retention (EPR effect), which has been becoming the gold standard for the design of tumor-targeted nanomedicine [5,6]. Along this line, he and his colleagues developed many polymer-based nanomedicines. One promising candidate drug is polymeric zinc protoporphyrin (P-ZnPP), which has been verified in many tumor models as having superior properties for tumor-targeted photodynamic therapy and diagnosis [7]. Another successful sample is HPMA copolymer conjugated pirarubicin with tumor environment responsive release profiles (P-THP) [8]. P-THP exhibits remarkable therapeutic effect with very few side effects, which has not only been demonstrated in animal tumor models, but also in a pilot clinical research study [9]. Recently, a polymeric nano-drug for Boron Neutron Capture Therapy (BNCT) utilizing multiple anticancer mechanisms has been developed by his excellent idea and under his supervision [10]. All the ideas of these nanomedicines are not only based on the extensive knowledge and sharp scientific insight, but also considering the needs of patients. To develop drugs and therapeutics that possess a remarkable safety profile in patients while exhibiting high efficacy, that could be produced at low cost as was his goal, we believe it was his goal that remains to inspire researchers on cancer, and it will come true in the near future.

Prof. Maeda was a scientist with wide interest and perspectives. Besides his achievement in nanomedicine, he was also the pioneer on the research of reactive oxygen species (ROS) and infectious, inflammatory diseases. He was the first to claim that ROS is the major causes of tissue damages and disease progression in viral infection [11], and developed therapeutics for viral infection using an antioxidative agent. This original idea is now also used for the control of COVID-19. Further, he and his colleagues clarified the mechanisms of ROS generation during infection and inflammation and first revealed the association of ROS generation with carcinogenesis [12], by which he won the Tomizo Yoshida Prize of Japanese Cancer Associate, which is the top award for cancer research in Japan. Based on this knowledge, antioxidant has been becoming a popular topic and an indispensable event in everyday life, for the prevention of cancer as well as many other diseases. In this regard, Prof. Maeda even developed an efficient method to prepare vegetable soup with high antioxidative potency, and his book on vegetable soup has become a best-seller recently.

Over his career, Prof. Maeda held the patents of 14 inventions, including SMANCS. With this small number of inventions, he led a wave of scientific research that keeps its momentum to date [13].

Prof. Maeda was an excellent mentor to young researchers. On personal accounts, working with Prof. Maeda was always challenging. He was able to push forward his students to the limits of their capabilities, both intellectually and technically. He was a caring person, and he insisted that all the students would have lunch in the laboratory with him every day, leading discussion that involved all life issues.

With him, we came to realize how to think big and push the limits of our ideas. He encouraged daring and unconventional ideas. Prof. Maeda will always be remembered for his forward thinking and for his contribution to the field of anticancer nanomedicine.

## Data Availability

Not applicable.
